# Crystal structure of the salt bis­(tri­ethano­lamine-κ^3^ *N*,*O*,*O*′)cobalt(II) bis­[2-(2-oxo-2,3-di­hydro-1,3-benzo­thia­zol-3-yl)acetate]

**DOI:** 10.1107/S2056989016002930

**Published:** 2016-02-24

**Authors:** Jamshid M. Ashurov, Nodira J. Obidova, Hudaybergen B. Abdireymov, Bakhtiyar T. Ibragimov

**Affiliations:** a Institute of Bioorganic Chemistry, Academy of Sciences of Uzbekistan, M. Ulugbek Str. 83, Tashkent 700125, Uzbekistan; b Karakalpak State University, Nukus str. Abdirova 1, Karakalpakstan 742012, Uzbekistan

**Keywords:** crystal structure, tri­ethano­lamine, α-(*N*-benzo­thia­zolin-2-one) acetic acid, hydrogen bonding

## Abstract

The reaction of 2-(2-oxo-2,3-di­hydro-1,3-benzo­thia­zol-3-yl)acetic acid (NBTA) and tri­ethano­lamine (TEA) with Co(NO_3_)_2_ results in the formation of the title complex, [Co(C_6_H_15_NO_3_)_2_](C_9_H_6_NO_3_S)_2_, which is formed as a result of the association of bis­(tri­ethano­lamine)­cobalt(II) and 2-(2-oxo-2,3-di­hydro-1,3-benzo­thia­zol-3-yl)acetate units. It crystallizes in the monoclinic centrosymmetric space group *P*2_1_/*c*, with the Co^II^ ion situated on an inversion centre. In the complex cation, the Co^II^ ion is octa­hedrally coordinated by two *N*,*O*,*O*′-tridentate TEA mol­ecules with a facial distribution and the N atoms in a *trans* arrangement. Two ethanol groups of each TEA mol­ecule form two five-membered chelate rings around the Co^II^ ion, while the third ethanol group does not coordinate to the metal. The free and coordinating hy­droxy groups of the TEA mol­ecules are involved in hydrogen bonding with the O atoms of NBTA anions, forming an infinite two-dimensional network extending parallel to the *bc* plane.

## Abstract 2

The reaction of 2-(2-oxo-2,3-di­hydro-1,3-benzo­thia­zol-3-yl)acetic acid (NBTA) and tri­ethano­lamine (TEA) with Co(NO_3_)_2_ results in the formation of the title complex. In the complex cation, the Co^II^ ion is octa­hedrally coordinated by two *N*,*O*,*O*′-tridentate TEA mol­ecules with a facial distribution and the N atoms in a *trans* arrangement.

## Chemical context   

Tri­ethano­lamine (TEA) is used as a corrosion inhibitor in metal-cutting fluids, as a curing agent for ep­oxy and rubber polymers, adhesives and anti­static agents and as a pharmaceutical inter­mediate and an ointment emulsifier *etc*. However, TEA is not a substance possessing a specific physiological action (Beyer *et al.*, 1983 ▸; Knaak *et al.*, 1997 ▸) with exception of its low anti­bacterial activity. Benzo­thia­zole is a precursor for rubber accelerators, a component of cyanine dyes, a slimicide in the paper and pulp industry, and is used in the production of certain fungicides, herbicides, anti­fungal agents and pharmaceuticals (Bellavia *et al.*, 2000 ▸; Seo *et al.*, 2000 ▸). The inter­action of metal ions with TEA results in the formation of complexes in which TEA demonstrates monodentate (Kumar *et al.*, 2014 ▸), bidentate (Kapteijn *et al.*, 1997 ▸), tridentate (Gao *et al.*, 2004 ▸; Ucar *et al.*, 2004 ▸; Topcu *et al.*, 2001 ▸; Krabbes *et al.*, 1999 ▸; Haukka *et al.*, 2005 ▸; Yeşilel *et al.*, 2004 ▸; Mirskova *et al.*, 2013 ▸) and tetra­dentate binding (Zaitsev *et al.*, 2014 ▸; Kazak *et al.*, 2003 ▸; Yilmaz *et al.*, 2004 ▸; Langley *et al.*, 2011 ▸; Rickard *et al.*, 1999 ▸; Maestri & Brown, 2004 ▸; Kovbasyuk *et al.*, 2001 ▸; Tudor *et al.*, 2001 ▸). In some complexes, TEA can show bridging properties (Atria *et al.*, 2015 ▸; Wittick *et al.*, 2006 ▸; Sharma *et al.*, 2014 ▸; Yang *et al.*, 2014 ▸; Funes *et al.*, 2014 ▸). Here, we report the synthesis and structure of the title compound, [Co(C_6_H_15_NO_3_)_2_](C_9_H_6_NO_3_S)_2_, (**I**). 
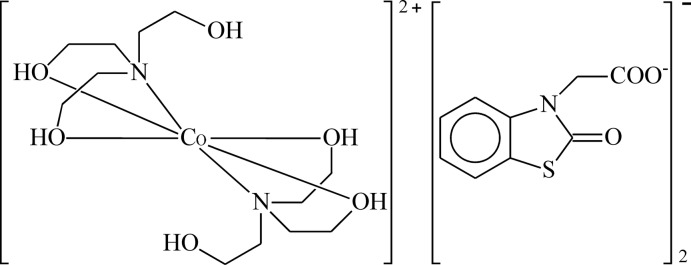


## Structural commentary   

**Figure 1:**
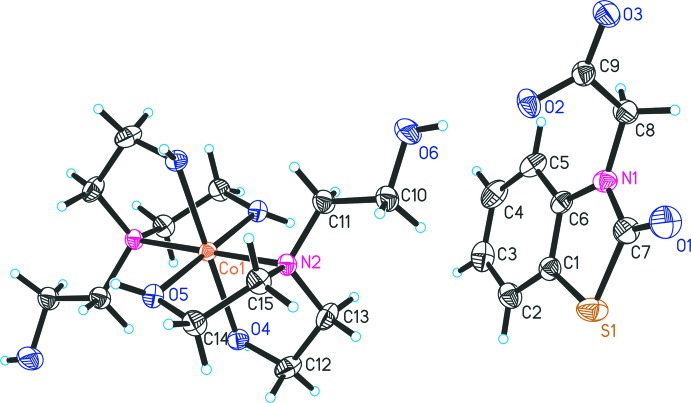
The mol­ecular structure of (**I**), showing the atom-labelling scheme. Unlabelled atoms are generated by the inversion centre.

The mol­ecular structure of compound (**I**) is shown in Fig. 1 ▸. The structure consists of a complex cation and one 2-(2-oxo-2,3-di­hydro-1,3-benzo­thia­zol-3-yl)acetate anion. The asymmetric unit contains a half of the cationic moiety because the Co^II^ ion is located on an inversion centre. The cation and anion are linked by an O6—H6⋯O2 hydrogen bond (Table 1 ▸). In the cationic complex, the Co^II^ ion is coordinated by four oxygen and two nitro­gen atoms of two ligands. The nitro­gen atoms occupy *trans* positions of the coordination polyhedron. The Co—N bond lengths [2.151 (3) Å] are equal as a result of symmetry, and the N—Co—N bond angle is 180°. The Co—O distances are 2.097 (2) Å and 2.101 (3) Å. One hy­droxy group of each ethanol substituent is not involved in the coordination and is directed away from the coordination centre. The N—Co—O bond angles range from 81.60 (10) to 98.40 (10)° and the O—Co—O angles are 89.79 (10) and 90.21 (10)°. Thus, the coordination polyhedron of the central atom is a slightly distorted octa­hedron of the CoN_2_O_4_-type. The thia­zoline ring (C1/C6/N1/C7/S1) and the bicyclic benzo­thia­zole unit (N1/S1/C1–C7) are close to planar, the largest deviations from the least-squares planes being 0.019 (2) and 0.028 (4) Å, respectively. The dihedral angle between the plane of the carboxyl­ate group and the benzo­thia­zole ring system is 85.6 (2)°.

## Supra­molecular features   

**Figure 2:**
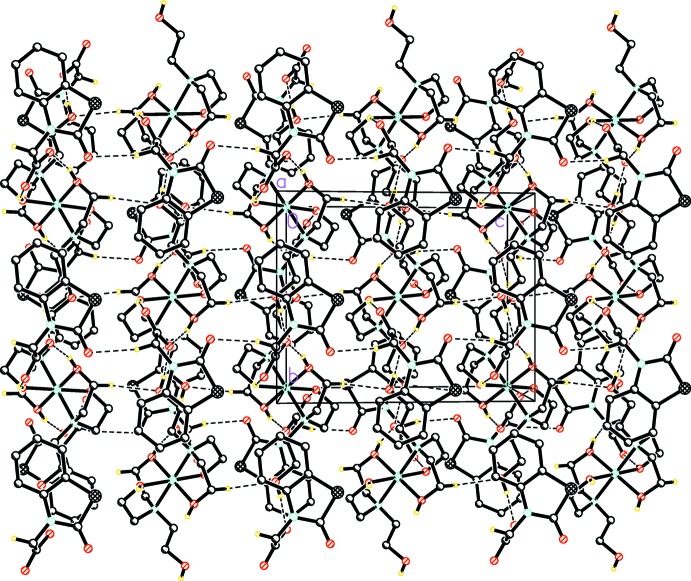
Part of the crystal structure with hydrogen bonds shown as dashed lines. For clarity, H atoms not involved in hydrogen bonding are not shown.

**Figure 3:**
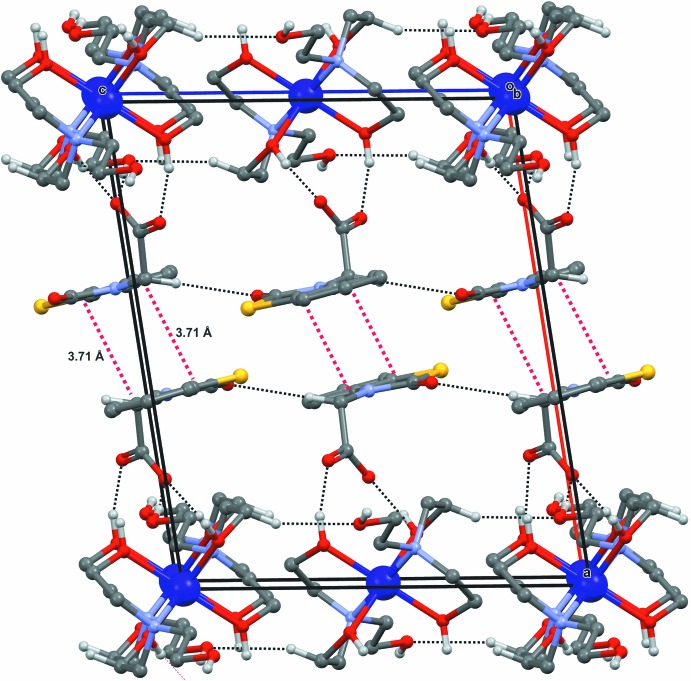
The crystal structure packing of (**I**). Hydrogen bonds are indicated by black dashed lines and π–π stacking inter­actions by red dashed lines.

The crystal structure of (**I**) contains an intricate network of inter­molecular O—H⋯O and C—H⋯O hydrogen bonds (Table 1 ▸). The [Co(TEA)_2_]^2+^ cations play an important role in the supra­molecular architecture. Each cation is surrounded by four 2-(2-oxo-2,3-di­hydro-1,3-benzo­thia­zol-3-yl)acetate anions. The H atoms of the free hy­droxy group of the TEA ligand form a hydrogen bond with the carboxyl­ate O atom of the NBTA ion while the coordinating hy­droxy H atoms are involved in inter­molecular hydrogen bonding with the carboxyl­ate O atoms of the NBTA ions [H4⋯O2^i^ = 1.71 (3) Å and H5⋯O3^ii^ = 1.752 (17)Å; symmetry codes: (i) *x*, −1 + *y*, z; (ii) 2 − *x*, 1 − *y*, 2 − *z*]. In addition, there is weak hydrogen bond between the –CH_2_ group and the non-coordinating hy­droxy-O atoms of the TEA ligand, with a C⋯O distance of 3.455 (6) Å. The above-mentioned hydrogen bonds give rise to 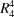
(22) and 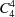
(22) graph-set motifs. The crystal structure contains layers of hydrogen-bonded cations that are sandwiched between layers of hydrogen-bonded anions. Each layer extends in the *bc* plane. There is hydrogen bonding within and between these layers. These are arranged along [100] in the sequence *ACA*·*ACA*·*ACA* (where *A* = anion layer and *C* = cation layer; Fig. 2 ▸) The NBTA anion layers are not linked by hydrogen bonds, but there are π–π stacking inter­actions between benzene (centroid *Cg*1) and thia­zolin (centroid *Cg*2) rings [*Cg*1⋯*Cg*2(-x, −y, −z) = 3.71 Å] of adjacent inversion-related mol­ecules (Fig. 3 ▸).

## Database survey   

A survey of the Cambridge Structural Database (CSD; Groom & Allen, 2014 ▸) showed that coordination complexes of TEA with many metals including those of the *s*-, *d*-, *p*-, and *f*-blocks have been reported. Structures containing the bis­(tri­ethano­lamine)­cobalt(II) cation are described in the CSD entries with refcodes ASUGEA, IGALOR, WEPLIN.

## Synthesis and crystallization   

To an aqueous solution (2.5 ml) of Co(NO_3_)_2_ (0.091 g, 0.5 mmol) was added slowly an ethanol solution (5 ml) containing TEA (132 µl) and NBTA (0.209 g, 1 mmol) with constant stirring. A light-brown crystalline product was obtained at room temperature by solvent evaporation after four weeks (yield 70%). Elemental analysis calculated for C_30_H_42_CoN_4_O_12_S_2_: C, 46.57; H, 5.47; N, 7.24. Found: C, 46.62; H, 5.41; N, 7.19.

## Refinement details   

Crystal data, data collection and structure refinement details are summarized in Table 2 ▸. The coordinating hy­droxy H atoms of the TEA ligand were located in a difference Fourier map and freely refined. C-bound H atoms were placed in calculated positions and refined as riding atoms: C—H = 0.93 and 0.97 Å for aromatic and methyl­ene H, with *U*_iso_(H) = 1.2*U*_eq_(C).

## Supplementary Material

Crystal structure: contains datablock(s) I. DOI: 10.1107/S2056989016002930/pj2027sup1.cif

Structure factors: contains datablock(s) I. DOI: 10.1107/S2056989016002930/pj2027Isup2.hkl

CCDC reference: 1454443

Additional supporting information:  crystallographic information; 3D view; checkCIF report

## Figures and Tables

**Table 1:** Hydrogen-bond geometry (Å, °)

*D*—H⋯*A*	*D*—H	H⋯*A*	*D*⋯*A*	*D*—H⋯*A*
O4—H4⋯O2^i^	0.88 (3)	1.71 (3)	2.572 (4)	166 (3)
O5—H5⋯O3^ii^	0.86 (1)	1.75 (2)	2.577 (4)	159 (3)
O6—H6⋯O2	0.82	1.88	2.697 (4)	173
C8—H8*A*⋯O1^iii^	0.97	2.48	3.432 (6)	167
C12—H12*B*⋯O6^iv^	0.97	2.53	3.455 (6)	159

Symmetry codes: (i) 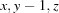; (ii) 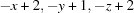; (iii) 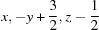; (iv) 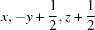.

**Table 2:** Experimental details

Crystal data	
Chemical formula	[Co(C_6_H_15_NO_3_)_2_](C_9_H_6_NO_3_S)_2_
*M*_r_	773.73
Crystal system, space group	Monoclinic, *P*2_1_/*c*
Temperature (K)	293
*a*, *b*, *c* (Å)	14.6953 (6), 9.7043 (3), 12.1311 (4)
β (°)	98.513 (4)
*V* (Å^3^)	1710.94 (11)
*Z*	2
Radiation type	Cu *K*α
μ (mm^−1^)	5.66
Crystal size (mm)	0.28 × 0.24 × 0.18

Data collection	
Diffractometer	Oxford Diffraction Xcalibur Ruby
Absorption correction	Multi-scan (SCALE3 ABSPACK in *CrysAlis PRO*; Oxford Diffraction, 2009 ▸)
*T*_min_, *T*_max_	0.280, 0.797
No. of measured, independent and observed [*I* > 2σ(*I*)] reflections	7096, 3487, 2693
*R*_int_	0.048
(sin θ/λ)_max_ (Å^−1^)	0.629

Refinement	
*R*[*F* ^2^ > 2σ(*F* ^2^)], *wR*(*F* ^2^), *S*	0.061, 0.175, 1.03
No. of reflections	3487
No. of parameters	230
No. of restraints	6
H-atom treatment	H atoms treated by a mixture of independent and constrained refinement
Δρ_max_, Δρ_min_ (e Å^−3^)	0.47, −0.53

Computer programs: *CrysAlis PRO* (Oxford Diffraction, 2009 ▸), *SHELXS97*, *SHELXL97* *XP* and *SHELXTL* (Sheldrick, 2008 ▸).

## supplementary crystallographic information

### Crystal data

**Table:**

[Co(C_6_H_15_NO_3_)_2_](C_9_H_6_NO_3_S)_2_	*F*(000) = 810
*M**_r_* = 773.73	*D*_x_ = 1.502 Mg m^−^^3^
Monoclinic, *P*2_1_/*c*	Cu *K*α radiation, λ = 1.54184 Å
Hall symbol: -P 2ybc	Cell parameters from 1414 reflections
*a* = 14.6953 (6) Å	θ = 3.7–75.3°
*b* = 9.7043 (3) Å	µ = 5.66 mm^−^^1^
*c* = 12.1311 (4) Å	*T* = 293 K
β = 98.513 (4)°	Block, dark orange
*V* = 1710.94 (11) Å^3^	0.28 × 0.24 × 0.18 mm
*Z* = 2	

### Data collection

**Table:**

Oxford Diffraction Xcalibur Ruby diffractometer	3487 independent reflections
Radiation source: fine-focus sealed tube	2693 reflections with *I* > 2σ(*I*)
Graphite monochromator	*R*_int_ = 0.048
Detector resolution: 10.2576 pixels mm^-1^	θ_max_ = 75.8°, θ_min_ = 5.5°
ω scans	*h* = −18→17
Absorption correction: multi-scan (SCALE3 ABSPACK in *CrysAlis PRO*; Oxford Diffraction, 2009)	*k* = −10→12
*T*_min_ = 0.280, *T*_max_ = 0.797	*l* = −12→15
7096 measured reflections	

### Refinement

**Table:**

Refinement on *F*^2^	Primary atom site location: structure-invariant direct methods
Least-squares matrix: full	Secondary atom site location: difference Fourier map
*R*[*F*^2^ > 2σ(*F*^2^)] = 0.061	Hydrogen site location: inferred from neighbouring sites
*wR*(*F*^2^) = 0.175	H atoms treated by a mixture of independent and constrained refinement
*S* = 1.03	*w* = 1/[σ^2^(*F*_o_^2^) + (0.0899*P*)^2^ + 0.4878*P*] where *P* = (*F*_o_^2^ + 2*F*_c_^2^)/3
3487 reflections	(Δ/σ)_max_ < 0.001
230 parameters	Δρ_max_ = 0.47 e Å^−^^3^
6 restraints	Δρ_min_ = −0.53 e Å^−^^3^

### Special details

**Table:**

Geometry. All e.s.d.'s (except the e.s.d. in the dihedral angle between two l.s. planes) are estimated using the full covariance matrix. The cell e.s.d.'s are taken into account individually in the estimation of e.s.d.'s in distances, angles and torsion angles; correlations between e.s.d.'s in cell parameters are only used when they are defined by crystal symmetry. An approximate (isotropic) treatment of cell e.s.d.'s is used for estimating e.s.d.'s involving l.s. planes.
Refinement. Refinement of *F*^2^ against ALL reflections. The weighted *R*-factor *wR* and goodness of fit *S* are based on *F*^2^, conventional *R*-factors *R* are based on *F*, with *F* set to zero for negative *F*^2^. The threshold expression of *F*^2^ > σ(*F*^2^) is used only for calculating *R*-factors(gt) *etc*. and is not relevant to the choice of reflections for refinement. *R*-factors based on *F*^2^ are statistically about twice as large as those based on *F*, and *R*- factors based on ALL data will be even larger.

### Fractional atomic coordinates and isotropic or equivalent isotropic displacement parameters (Å^2^)

**Table:**

	*x*	*y*	*z*	*U*_iso_*/*U*_eq_	
Co1	1.0000	0.0000	1.0000	0.0321 (2)	
S1	1.42643 (10)	0.51313 (12)	1.23063 (9)	0.0613 (3)	
O4	1.09939 (16)	−0.1150 (3)	1.1029 (2)	0.0405 (6)	
H4	1.1359 (16)	−0.175 (3)	1.078 (2)	0.061*	
O2	1.21319 (18)	0.7409 (3)	1.0090 (3)	0.0511 (7)	
O5	0.92168 (17)	0.0172 (2)	1.1309 (2)	0.0397 (6)	
H5	0.8630 (7)	0.025 (4)	1.130 (2)	0.060*	
C4	1.3618 (3)	0.3107 (6)	0.8983 (4)	0.0640 (12)	
H4A	1.3469	0.2728	0.8275	0.077*	
N2	1.07355 (19)	0.1681 (3)	1.0879 (2)	0.0354 (6)	
O6	1.1297 (3)	0.4967 (3)	0.9585 (3)	0.0719 (11)	
H6	1.1537	0.5703	0.9793	0.108*	
O3	1.25064 (18)	0.9228 (3)	0.9142 (3)	0.0593 (8)	
O1	1.4058 (3)	0.7837 (3)	1.1993 (3)	0.0715 (9)	
N1	1.3885 (2)	0.6444 (3)	1.0445 (3)	0.0432 (7)	
C11	1.0861 (3)	0.2771 (4)	1.0053 (3)	0.0455 (9)	
H11A	1.0256	0.3054	0.9693	0.055*	
H11B	1.1176	0.2363	0.9484	0.055*	
C1	1.4051 (2)	0.4195 (4)	1.1068 (3)	0.0441 (8)	
C9	1.2684 (2)	0.8119 (4)	0.9634 (3)	0.0437 (8)	
C10	1.1382 (3)	0.4043 (4)	1.0483 (3)	0.0507 (9)	
H10A	1.1122	0.4435	1.1102	0.061*	
H10B	1.2024	0.3826	1.0731	0.061*	
C6	1.3850 (2)	0.5060 (4)	1.0156 (3)	0.0405 (8)	
C13	1.1640 (3)	0.1118 (4)	1.1391 (4)	0.0535 (10)	
H13A	1.1899	0.1712	1.2002	0.064*	
H13B	1.2056	0.1126	1.0841	0.064*	
C15	1.0160 (3)	0.2213 (4)	1.1688 (3)	0.0464 (9)	
H15A	0.9732	0.2890	1.1321	0.056*	
H15B	1.0551	0.2672	1.2292	0.056*	
C8	1.3671 (3)	0.7592 (4)	0.9693 (4)	0.0500 (10)	
H8A	1.3771	0.7314	0.8952	0.060*	
H8B	1.4092	0.8341	0.9928	0.060*	
C7	1.4049 (3)	0.6711 (5)	1.1570 (4)	0.0520 (10)	
C12	1.1576 (3)	−0.0320 (5)	1.1821 (4)	0.0542 (11)	
H12A	1.2186	−0.0725	1.1962	0.065*	
H12B	1.1329	−0.0298	1.2520	0.065*	
C3	1.3811 (3)	0.2232 (5)	0.9886 (4)	0.0628 (12)	
H3	1.3783	0.1283	0.9781	0.075*	
C5	1.3638 (3)	0.4522 (5)	0.9094 (3)	0.0522 (10)	
H5A	1.3512	0.5095	0.8475	0.063*	
C2	1.4045 (3)	0.2764 (4)	1.0941 (4)	0.0540 (10)	
H2	1.4195	0.2186	1.1552	0.065*	
C14	0.9626 (3)	0.1087 (4)	1.2162 (3)	0.0480 (9)	
H14A	1.0035	0.0572	1.2714	0.058*	
H14B	0.9148	0.1495	1.2530	0.058*	

### Atomic displacement parameters (Å^2^)

**Table:**

	*U*^11^	*U*^22^	*U*^33^	*U*^12^	*U*^13^	*U*^23^
Co1	0.0309 (4)	0.0303 (4)	0.0344 (4)	0.0010 (3)	0.0022 (3)	0.0001 (3)
S1	0.0879 (9)	0.0538 (7)	0.0408 (5)	0.0058 (5)	0.0049 (5)	0.0012 (4)
O4	0.0378 (12)	0.0369 (14)	0.0464 (13)	0.0066 (10)	0.0050 (10)	0.0020 (11)
O2	0.0412 (13)	0.0348 (14)	0.0799 (19)	0.0011 (11)	0.0180 (13)	0.0036 (13)
O5	0.0371 (12)	0.0378 (14)	0.0445 (14)	−0.0006 (10)	0.0072 (10)	−0.0045 (10)
C4	0.056 (2)	0.075 (3)	0.058 (3)	0.007 (2)	−0.001 (2)	−0.025 (2)
N2	0.0368 (14)	0.0308 (15)	0.0384 (14)	−0.0032 (11)	0.0050 (11)	−0.0025 (12)
O6	0.090 (3)	0.048 (2)	0.072 (2)	−0.0219 (16)	−0.007 (2)	0.0152 (16)
O3	0.0405 (14)	0.0499 (18)	0.089 (2)	0.0076 (13)	0.0143 (14)	0.0197 (16)
O1	0.094 (3)	0.0485 (19)	0.069 (2)	0.0017 (17)	0.0040 (18)	−0.0165 (16)
N1	0.0388 (15)	0.0467 (19)	0.0439 (16)	0.0064 (13)	0.0056 (12)	0.0039 (14)
C11	0.052 (2)	0.039 (2)	0.0442 (19)	−0.0072 (17)	0.0051 (16)	−0.0020 (16)
C1	0.0381 (17)	0.048 (2)	0.046 (2)	0.0000 (16)	0.0090 (15)	−0.0006 (17)
C9	0.0375 (17)	0.041 (2)	0.052 (2)	0.0019 (15)	0.0066 (15)	−0.0002 (17)
C10	0.057 (2)	0.043 (2)	0.051 (2)	−0.0116 (18)	0.0041 (18)	−0.0016 (18)
C6	0.0294 (15)	0.050 (2)	0.0422 (19)	0.0057 (14)	0.0044 (14)	−0.0037 (16)
C13	0.041 (2)	0.049 (2)	0.066 (2)	−0.0023 (17)	−0.0108 (18)	−0.012 (2)
C15	0.055 (2)	0.040 (2)	0.045 (2)	−0.0034 (17)	0.0101 (17)	−0.0086 (17)
C8	0.0390 (18)	0.051 (2)	0.062 (2)	0.0061 (17)	0.0114 (17)	0.011 (2)
C7	0.054 (2)	0.047 (2)	0.054 (2)	0.0028 (18)	0.0059 (18)	−0.003 (2)
C12	0.055 (2)	0.048 (2)	0.053 (2)	0.0105 (18)	−0.0121 (19)	−0.0029 (19)
C3	0.052 (2)	0.051 (3)	0.086 (3)	0.000 (2)	0.014 (2)	−0.017 (2)
C5	0.046 (2)	0.066 (3)	0.043 (2)	0.013 (2)	0.0007 (17)	−0.009 (2)
C2	0.053 (2)	0.044 (2)	0.067 (3)	0.0011 (18)	0.014 (2)	0.003 (2)
C14	0.056 (2)	0.048 (2)	0.0405 (19)	−0.0068 (18)	0.0112 (16)	−0.0064 (17)

### Geometric parameters (Å, º)

**Table:**

Co1—O4	2.097 (2)	C11—C10	1.505 (5)
Co1—O4^i^	2.097 (2)	C11—H11A	0.9700
Co1—O5^i^	2.101 (3)	C11—H11B	0.9700
Co1—O5	2.101 (3)	C1—C6	1.386 (5)
Co1—N2^i^	2.151 (3)	C1—C2	1.398 (6)
Co1—N2	2.151 (3)	C9—C8	1.529 (5)
S1—C1	1.744 (4)	C10—H10A	0.9700
S1—C7	1.779 (5)	C10—H10B	0.9700
O4—C12	1.436 (5)	C6—C5	1.382 (5)
O4—H4	0.875 (9)	C13—C12	1.498 (6)
O2—C9	1.254 (4)	C13—H13A	0.9700
O5—C14	1.427 (4)	C13—H13B	0.9700
O5—H5	0.863 (9)	C15—C14	1.509 (5)
C4—C5	1.380 (7)	C15—H15A	0.9700
C4—C3	1.382 (7)	C15—H15B	0.9700
C4—H4A	0.9300	C8—H8A	0.9700
N2—C15	1.480 (4)	C8—H8B	0.9700
N2—C13	1.486 (5)	C12—H12A	0.9700
N2—C11	1.487 (5)	C12—H12B	0.9700
O6—C10	1.401 (5)	C3—C2	1.374 (6)
O6—H6	0.8200	C3—H3	0.9300
O3—C9	1.239 (5)	C5—H5A	0.9300
O1—C7	1.207 (5)	C2—H2	0.9300
N1—C7	1.374 (5)	C14—H14A	0.9700
N1—C6	1.387 (5)	C14—H14B	0.9700
N1—C8	1.445 (5)		
			
O4—Co1—O4^i^	179.999 (1)	C11—C10—H10A	110.6
O4—Co1—O5^i^	89.79 (10)	O6—C10—H10B	110.6
O4^i^—Co1—O5^i^	90.21 (10)	C11—C10—H10B	110.6
O4—Co1—O5	90.21 (10)	H10A—C10—H10B	108.7
O4^i^—Co1—O5	89.79 (10)	C5—C6—C1	120.5 (4)
O5^i^—Co1—O5	180.00 (14)	C5—C6—N1	126.6 (4)
O4—Co1—N2^i^	98.40 (10)	C1—C6—N1	112.9 (3)
O4^i^—Co1—N2^i^	81.60 (10)	N2—C13—C12	112.9 (3)
O5^i^—Co1—N2^i^	81.74 (10)	N2—C13—H13A	109.0
O5—Co1—N2^i^	98.26 (10)	C12—C13—H13A	109.0
O4—Co1—N2	81.60 (10)	N2—C13—H13B	109.0
O4^i^—Co1—N2	98.40 (10)	C12—C13—H13B	109.0
O5^i^—Co1—N2	98.26 (10)	H13A—C13—H13B	107.8
O5—Co1—N2	81.74 (10)	N2—C15—C14	112.4 (3)
N2^i^—Co1—N2	180.0	N2—C15—H15A	109.1
C1—S1—C7	91.15 (19)	C14—C15—H15A	109.1
C12—O4—Co1	113.2 (2)	N2—C15—H15B	109.1
C12—O4—H4	105.5 (16)	C14—C15—H15B	109.1
Co1—O4—H4	124.2 (16)	H15A—C15—H15B	107.9
C14—O5—Co1	112.2 (2)	N1—C8—C9	113.8 (3)
C14—O5—H5	105.9 (15)	N1—C8—H8A	108.8
Co1—O5—H5	130.5 (18)	C9—C8—H8A	108.8
C5—C4—C3	122.3 (4)	N1—C8—H8B	108.8
C5—C4—H4A	118.8	C9—C8—H8B	108.8
C3—C4—H4A	118.8	H8A—C8—H8B	107.7
C15—N2—C13	114.6 (3)	O1—C7—N1	125.6 (4)
C15—N2—C11	109.8 (3)	O1—C7—S1	125.2 (3)
C13—N2—C11	110.5 (3)	N1—C7—S1	109.2 (3)
C15—N2—Co1	107.4 (2)	O4—C12—C13	110.6 (3)
C13—N2—Co1	106.3 (2)	O4—C12—H12A	109.5
C11—N2—Co1	108.0 (2)	C13—C12—H12A	109.5
C10—O6—H6	109.5	O4—C12—H12B	109.5
C7—N1—C6	115.3 (3)	C13—C12—H12B	109.5
C7—N1—C8	118.2 (3)	H12A—C12—H12B	108.1
C6—N1—C8	126.1 (3)	C2—C3—C4	120.1 (5)
N2—C11—C10	117.2 (3)	C2—C3—H3	120.0
N2—C11—H11A	108.0	C4—C3—H3	120.0
C10—C11—H11A	108.0	C4—C5—C6	117.7 (4)
N2—C11—H11B	108.0	C4—C5—H5A	121.1
C10—C11—H11B	108.0	C6—C5—H5A	121.1
H11A—C11—H11B	107.2	C3—C2—C1	118.2 (4)
C6—C1—C2	121.1 (4)	C3—C2—H2	120.9
C6—C1—S1	111.2 (3)	C1—C2—H2	120.9
C2—C1—S1	127.6 (3)	O5—C14—C15	111.2 (3)
O3—C9—O2	125.8 (3)	O5—C14—H14A	109.4
O3—C9—C8	116.4 (3)	C15—C14—H14A	109.4
O2—C9—C8	117.8 (3)	O5—C14—H14B	109.4
O6—C10—C11	105.9 (3)	C15—C14—H14B	109.4
O6—C10—H10A	110.6	H14A—C14—H14B	108.0
			
O4^i^—Co1—O4—C12	−139 (11)	C2—C1—C6—N1	179.6 (3)
O5^i^—Co1—O4—C12	103.4 (3)	S1—C1—C6—N1	1.1 (4)
O5—Co1—O4—C12	−76.6 (3)	C7—N1—C6—C5	175.5 (4)
N2^i^—Co1—O4—C12	−175.0 (3)	C8—N1—C6—C5	2.5 (6)
N2—Co1—O4—C12	5.0 (3)	C7—N1—C6—C1	−4.1 (5)
O4—Co1—O5—C14	72.2 (2)	C8—N1—C6—C1	−177.1 (3)
O4^i^—Co1—O5—C14	−107.8 (2)	C15—N2—C13—C12	80.4 (4)
O5^i^—Co1—O5—C14	−141.4 (8)	C11—N2—C13—C12	−154.9 (3)
N2^i^—Co1—O5—C14	170.7 (2)	Co1—N2—C13—C12	−38.0 (4)
N2—Co1—O5—C14	−9.3 (2)	C13—N2—C15—C14	−83.1 (4)
O4—Co1—N2—C15	−105.4 (2)	C11—N2—C15—C14	151.8 (3)
O4^i^—Co1—N2—C15	74.6 (2)	Co1—N2—C15—C14	34.7 (4)
O5^i^—Co1—N2—C15	166.0 (2)	C7—N1—C8—C9	−77.0 (5)
O5—Co1—N2—C15	−14.0 (2)	C6—N1—C8—C9	95.8 (4)
N2^i^—Co1—N2—C15	−5 (14)	O3—C9—C8—N1	169.0 (4)
O4—Co1—N2—C13	17.6 (2)	O2—C9—C8—N1	−10.3 (5)
O4^i^—Co1—N2—C13	−162.4 (2)	C6—N1—C7—O1	−176.0 (4)
O5^i^—Co1—N2—C13	−70.9 (3)	C8—N1—C7—O1	−2.4 (6)
O5—Co1—N2—C13	109.1 (3)	C6—N1—C7—S1	4.9 (4)
N2^i^—Co1—N2—C13	118 (14)	C8—N1—C7—S1	178.5 (3)
O4—Co1—N2—C11	136.3 (2)	C1—S1—C7—O1	177.4 (4)
O4^i^—Co1—N2—C11	−43.7 (2)	C1—S1—C7—N1	−3.5 (3)
O5^i^—Co1—N2—C11	47.7 (2)	Co1—O4—C12—C13	−26.9 (4)
O5—Co1—N2—C11	−132.3 (2)	N2—C13—C12—O4	44.2 (5)
N2^i^—Co1—N2—C11	−123 (13)	C5—C4—C3—C2	−0.9 (7)
C15—N2—C11—C10	64.6 (4)	C3—C4—C5—C6	−0.7 (7)
C13—N2—C11—C10	−62.7 (4)	C1—C6—C5—C4	1.1 (6)
Co1—N2—C11—C10	−178.6 (3)	N1—C6—C5—C4	−178.4 (4)
C7—S1—C1—C6	1.4 (3)	C4—C3—C2—C1	2.0 (6)
C7—S1—C1—C2	−177.0 (4)	C6—C1—C2—C3	−1.6 (6)
N2—C11—C10—O6	−172.3 (4)	S1—C1—C2—C3	176.6 (3)
C2—C1—C6—C5	0.1 (6)	Co1—O5—C14—C15	30.8 (4)
S1—C1—C6—C5	−178.4 (3)	N2—C15—C14—O5	−44.5 (4)

Symmetry code: (i) −*x*+2, −*y*, −*z*+2.

### Hydrogen-bond geometry (Å, º)

**Table:**

*D*—H···*A*	*D*—H	H···*A*	*D*···*A*	*D*—H···*A*
O4—H4···O2^ii^	0.88 (3)	1.71 (3)	2.572 (4)	166 (3)
O5—H5···O3^iii^	0.86 (1)	1.75 (2)	2.577 (4)	159 (3)
O6—H6···O2	0.82	1.88	2.697 (4)	173
C8—H8*A*···O1^iv^	0.97	2.48	3.432 (6)	167
C12—H12*B*···O6^v^	0.97	2.53	3.455 (6)	159

Symmetry codes: (ii) *x*, *y*−1, *z*; (iii) −*x*+2, −*y*+1, −*z*+2; (iv) *x*, −*y*+3/2, *z*−1/2; (v) *x*, −*y*+1/2, *z*+1/2.
